# Identification of RACK1 as a novel regulator of non-structural protein 4 of chikungunya virus: RACK1 regulates CHIKV nsP4

**DOI:** 10.3724/abbs.2024073

**Published:** 2024-05-29

**Authors:** Yao Yan, Fengyuan Zhang, Meng Zou, Hongyu Chen, Jingwen Xu, Shuaiyao Lu, Hongqi Liu

**Affiliations:** 1 Institute of Medical Biology Chinese Academy of Medical Sciences and Peking Union Medical College Kunming 650118 China; 2 National Human Diseases Animal Model Resource Center NHC Key Laboratory of Human Disease Comparative Medicine National Center of Technology Innovation for Animal Model State Key Laboratory of Respiratory Health and Multimorbidity and Key Laboratory of Pathogen Infection Prevention and Control Ministry of Education Institute of Laboratory Animal Sciences Chinese Academy of Medical Sciences and Peking Union Medical College Beijing 100021 China

**Keywords:** Chikungunya virus, non-structural protein 4, RACK1, ubiquitin-proteasome pathway

## Abstract

Chikungunya virus (CHIKV) is a neglected arthropod-borne and anthropogenic alphavirus. Over the past two decades, the CHIKV distribution has undergone significant changes worldwide, from the original tropics and subtropics regions to temperate regions, which has attracted global attention. However, the interactions between CHIKV and its host remain insufficiently understood, which dampens the need for the development of an anti-CHIKV strategy. In this study, on the basis of the optimal overexpression of non-structural protein 4 (nsP4), we explore host interactions of CHIKV nsP4 using mass spectrometry-based protein-protein interaction approaches. The results reveal that some cellular proteins that interact with nsP4 are enriched in the ubiquitin-proteasome pathway. Specifically, the scaffold protein receptor for activated C kinase 1 (RACK1) is identified as a novel host interactor and regulator of CHIKV nsP4. The inhibition of the interaction between RACK1 and nsP4 by harringtonolide results in the reduction of nsP4, which is caused by the promotion of degradation but not the inhibition of nsP4 translation. Furthermore, the decrease in nsP4 triggered by the RACK1 inhibitor can be reversed by the proteasome inhibitor MG132, suggesting that RACK1 can protect nsP4 from degradation through the ubiquitin-proteasome pathway. This study reveals a novel mechanism by which the host factor RACK1 regulates CHIKV nsP4, which could be a potential target for developing drugs against CHIKV.

## Introduction

Chikungunya virus (CHIKV) is a single positive-sense RNA virus transmitted by mosquitoes and belongs to the *Alphavirus* genus of the family *Togaviridae* [1]. The disease caused by CHIKV infection is referred to as chikungunya fever (CHIKF), characterized by sudden fever, rash, myalgia, and other syndromes [2]. Myalgia and arthritis symptoms can persist for months to years [3], which may cause severe spiritual and economic burdens on patients. CHIKV was first reported in Tanzania, a country in Africa, in 1952 when CHIKV was initially limited to local transmission [ 4, 5] . However, since 2004, CHIKV has unexpectedly emerged on the Indian Ocean islands due to adaptive mutations (alanine-to-valine substitution at position 226 of the E1 protein) [ 6, 7] . This mutation significantly increases CHIKV infectivity, leading to the rapid occurrence of large-scale epidemics [ 8, 9] . Global warming, coupled with the development of transportation and tourism, has further accelerated the global spread of CHIKV. To date, over 100 countries have reported outbreaks of CHIKV infection [10]. According to phylogenetic analysis, CHIKV can be divided into four different genotypes, namely, West African (WA), East-Central-South African (ECSA), Asian and Indian Ocean (IOL), corresponding to their respective geographical origins [5].

The CHIKV genome consists of a capped 5′-UTR, two open reading frames (ORFs), and a 3′-UTR poly(A). ORF1 at the 5′ terminus encodes a nonstructural polyprotein that is catalyzed into four individual nsPs, named as nsP1, nsP2, nsP3 and nsP4. The ORF2 at the 3′ terminus encodes a structural polyprotein that is ultimately cleaved into 5‒6 structural proteins, including capsid protein (C), 3 envelope proteins (E1, E2, and E3), 6K and TF [11]. To facilitate CHIKV replication, these viral proteins cooperate and function at various stages of the viral life cycle. In addition, they also interact with host proteins and hijack a significant number of host proteins to participate in cellular pathways for viral replication [ 12, 13] . Non-structural proteins are first translated viral proteins from CHIKV RNA after entry into the cytosol of infected cells and then participate in the synthesis of the viral genome and subgenome. RNA-dependent RNA polymerase (RdRp) is the first essential enzyme for viral RNA synthesis. Therefore, CHIKV nsP4 with RdRp activity is first cleaved from ns-polyprotein by the protease nsP2 and subsequently complexed with P123 to form a replicase for negative-strand RNA synthesis using viral positive RNA as a template. NsP1 is then cleaved by nsP2 and forms the short-lived complex nsP1+P23+nsP4. The mature viral replication complex (vRC) is formed by four fully processed nonstructural proteins and is responsible for the synthesis of the viral genome and subgenome RNA based on the negative RNA template. NsP1 is essential for viral RNA capping through its two enzymatic activities, N7-guanine-methyl-transferase (MTase) and guanylytransferase (GTase) that are critical for viral replication [14]. In addition, nsP1 possesses a membrane-binding peptide (aa 244‒263) that interacts with the cytoplasmic side of the cell bilayer membrane, by which nsP1 can transport vRC to the membrane to form a spherule that is a sequestered and safe site for RNA synthesis [ 15, 16] . NsP2 participates in viral RNA synthesis and capping via its helicase/NTPase and RNA phosphatase activities in addition to its protease activity, which mediates the production of individual mature nsPs from polyproteins [17]. NsP3 has three distinguishable domains: the most conserved macrodomain at the N-terminus, the unique middle alphavirus domain, and the C-terminal hypervariable domain (HAV). NsP3 acts as a hub for interactions with viral nsPs to form vRC in addition to the (ADP-ribosyl)hydrolase activity that is critical for the initiation of virus replication [18]. In addition to complexed nsPs in vRC, there are free nsPs in the cytoplasm or nucleus that may interact with host proteins to modulate the intracellular microenvironment [ 19, 20] . NsP1 mainly interacts with the cytoplasmic surface of the cell membrane, which is involved in the formation of spherules [21]. There are very few known host proteins that interact with nsP1. However, nsP1 downregulates the expression of the virus restriction factor BST2 via an unknown mechanism and possibly promotes viral release [22]. Co-IP and yeast two-hybrid assays revealed that nsP2 interacts with the human autophagy receptor NDP52, which enhances viral replication [23]. NsP3 directly interacts with G3BP1 and G3BP2 to prevent the formation of stress granules and consequently promotes viral RNA translation [24].

It is very challenging to explore CHIKV nsP4 due to its unique properties, including low abundance, instability and insolubility of the full-length protein in prokaryotic cells. Therefore, the majority of knowledge about CHIKV nsP4 is limited to other alphaviruses, particularly the less pathogenic Sindbis virus (SINV) and Semliki Forest virus (SFV) [ 25, 26] . Overexpression of EGFP-nsP4 revealed that CHIKV nsP4 inhibits tunicamycin-induced phosphorylation of eIF2α, which has also been observed in CHIKV-infected cells [27]. Furthermore, overexpression of EGFP-nsP4 resulted in the identification of a host interactor, Hsp90A. The interaction of CHIKV nsP4 with Hsp90A plays essential roles in the assembly of the viral replication complex [28]. These studies suggested that for investigations of nsP4-associated host factors and cellular events, overexpression of CHIKV nsP4 in cells may partially compensate for this shortage due to the low abundance of viral nsP4 in CHIKV-infected cells. More CHIKV nsP4 interactors remain to be identified, which is significant for the research and development of anti-CHIKV strategies.

In this study, via the overexpression of Asian CHIKV nsP4 in 293T cells, we identified a host interactor RACK1 that prevents the degradation of nsP4 via the ubiquitin-proteasome pathway. Therefore, RACK1, as a novel interactor of CHIKV nsP4, is a potential target for the R&D of drugs against CHIKV.

## Materials and Methods

### Cells and cell culture

293T cells (Procell, Shanghai, China) were grown and maintained at 37°C in a humidified incubator with a 5% CO _2_ atmosphere. The complete medium for the 293T cells was Dulbecco’s modified Eagle’s medium (DMEM; Corning, New York, USA) supplemented with 4.5 g/L glucose, 584 mg/L L-glutamine, and 110 mg/L sodium pyruvate, 10% fetal bovine serum (FBS; Gibco, Carlsbad,, USA), 100 U/mL penicillin, and 100 μg/mL streptomycin (Biological Industries, Beit Haemek, Israel).

### Antibodies and inhibitors

The antibodies used in this study included anti-nsP4 IgG (PA5-117443, rabbit; Invitrogen, Carlsbad, USA), anti-β-actin IgG (4970S, rabbit; Cell Signaling Technology, Beverly, USA), anti-phospho (Ser51) eIF2α IgG (3398S, rabbit; Cell Signaling Technology), anti-eIF2α IgG (5324S, rabbit; Cell Signaling Technology), anti-Hsp90 IgG (ab203126, rabbit; Abcam, Cambridge, USA), anti-GST IgG (11213-MM01, mouse; Sino Biological, Beijing, China), anti-RACK1 IgG (5432S, rabbit; Cell Signaling Technology), and anti-ubiquitin IgG (ab134953, rabbit; Abcam). The secondary antibodies included HRP-conjugated AffiniPure goat anti-rabbit IgG (H+L) (SA00001-2; Proteintech, Wuhan, China) and HRP-conjugated AffiniPure goat anti-mouse IgG (H+L) (SA00001-1; Proteintech). MG132 (HY-13259), Harringtonolide (HY-N10335), and cycloheximide (HY-12320) were purchased from MedChemExpress (MCE, Monmouth Junction, USA).

### CHIKV nsP4 and RdRp expression in 293T cells and *Escherichia coli*

The gene fragment of CHIKV nsP4 was amplified from the genomic RNA of the CHIKV Asian strain (GenBank OK316992), and then cloned into the mammalian expression vector using In-Fusion Snap Assembly Master Mix (TaKaRa, Shiga, Japan). The coding sequence for nsP4 RdRp was optimized and chemically synthesized by GENEWIZ (Suzhou, China), and inserted into the eukaryotic expression vector pMD2.GΔVSV (the original plasmid pMD2.G after the removal of *VSV* gene was used as the carrier plasmid) or prokaryotic expression vector pGEX-6P-1 using In-Fusion Snap Assembly Master Mix (TaKaRa, Dalian, China).

CHIKV nsP4 was expressed in 6-well plates. A total of 1×10 ^6^ 293T cells were seeded into each well of a 6-well plate and incubated overnight at 37°C in an incubator supplemented with 5% CO _2_. When the cells reached 80% confluence, they were transfected with 2.5 μg of recombinant DNA using a Lipofectamine 3000 Transfection kit (Thermo Fisher Scientific, Waltham, USA) according to the manufacturer’s protocol. At the designated time points, cell lysates were harvested for further analysis of nsP4 expression.

The expression of the prokaryotic expression vector pGEX-6P-1 with a GST tag in *E*. *coli* BL21(DE3) cells was induced by IPTG at a final concentration of 0.5 mM. The GST-RdRp fusion protein was purified using a glutathione high-speed chromatographic medium (4FF) preloaded column on the AKTA FPLC system as described in the manufacturer’s protocol. The purified GST-RdRp fusion protein was characterized by SDS-PAGE via Coomassie blue staining and western blot analysis with an anti-GST antibody. The concentration of the purified protein was determined on the basis of the standard protein BSA in SDS-PAGE, as described in the following section.

### SDS-PAGE and western blot analysis

A certain amount of protein with loading buffer was separated by 12% SDS-PAGE, followed by Coomassie blue staining, silver staining via the Protein Stains Q kit (Sangon Biotech, Shanghai, China) or western blot analysis as described below.

For western blot analysis, proteins were separated by 12% SDS-PAGE and then transferred onto methanol-treated PVDF membranes (Millipore, Billerica, USA). Five percent skim milk in PBST was used to block the PVDF membrane for at least 1 h at room temperature. After being washed once with PBST, the blocked PVDF membrane was incubated with primary antibody diluted in 2.5% PBST-BSA at 4°C overnight. After three times wash (10 min each), the PVDF membrane was incubated with an HRP-conjugated anti-IgG (H+L) antibody diluted with 2.5% skim milk in PBST at 37°C for 1 h. Then, the PVDF membrane was washed three times (10 min each) with PBST and developed with a West Pico Plus Chemiluminescent Substrate kit (Sagecreation, Beijing, China). Finally, images of the protein bands were captured with a ChampChemi® 580 gel imaging and analysis system (Sagecreation). For quantitative analysis, the intensity of each specific band was determined via ImageJ software (NIH, Bethesda, USA).

### Co-immunoprecipitation (Co-IP) analysis

Co-IP was performed using the Flag-tag Protein IP Assay Kit with Magnetic Beads (Beyotime, Shanghai, China) according to the manufacturer’s protocol. Briefly, a proper volume of magnetic beads was prepared, and the beads were washed twice with TBS on a magnetic rack. The cell lysate was prepared by resuspending the cell pellet in 150 μL of IP lysis solution and incubating on ice for 10 min, followed by centrifugation at 12,000 *g* for 10 min. The supernatant was collected and incubated with BeyoMag ^TM^ Mouse IgG Magnetic Beads in a rotating mixer at 4°C for 1 h to remove nonspecific proteins. The mixture was separated on a magnetic rack, and the supernatant was ready for the Co-IP experiment. BeyoMag ^TM^ Anti-Flag Magnetic Beads were added to the supernatant at a ratio of 20 μL of magnetic beads per 500 μL of lysate, which was incubated on a rotating mixer at 4°C overnight. The next day, the mixture was separated on a magnetic rack, and the supernatant was discarded. The magnetic beads were washed three times with 500 μL of IP cracking solution to remove nonspecifically bound proteins. Then, acid elution buffer was added to the magnetic beads (100 μL of acid elution buffer per 20 μL of original magnetic beads) to elute co-immunoprecipitated proteins by incubation at room temperature for 5 min on a rotating mixer. The mixture was separated on a magnetic rack for 10 s, and the supernatant was transferred to a new Eppendorf tube. Neutralization buffer (10 μL) was immediately added to the tube, and the co-immunoprecipitated proteins were ready for further analysis.

### GST pull-down experiment

A GST pull-down experiment was conducted using BeyoGold™ GST-tag Purification Resin (Beyotime) according to the recommended protocol. Briefly, an appropriate amount of 50% gel suspension was transferred to a centrifuge tube (50 μL of 50% gel suspension for each sample). GST pull-down protein binding buffer (20 times the volume of 50% gel suspension) was used to wash and equilibrate the resin. After removal of the supernatant, the remaining resin was resuspended in an equal volume of GST pull-down protein binding buffer. For each reaction, 400 μL of GST pull-down protein binding buffer, 50 μL of equilibrated 50% resin suspension, 25 μg of cell lysate or 25 μg of purified RACK1 protein (HY-P74606; MCE), and 25 μg of GST-tagged RdRp protein (decoy protein) or GST protein (negative control) were added to an Eppendorf tube, supplemented with GST pull-down protein binding buffer to make the same volume of each sample (0.5‒1 mL). After mixing, the tube was placed on a side shaker and incubated at 4°C overnight. The mixture was centrifuged at 1000 *g* at 4°C for 2 min. Then, the resin for each reaction was resuspended in 100 μL of GST pull-down protein binding buffer and centrifuged at 4°C (1000 *g* for 10 s) to fully wash off the unbound protein, which was repeated twice. Finally, 50 μL of GST elution buffer was used to resuspend the resin for each reaction, which was incubated for 10 min, followed by centrifugation at 1000 *g* at 4°C for 2 min. The proteins pulled down by the GST-RdRp fusion protein in the supernatant were harvested for subsequent analysis.

### Mass spectrometry analysis

Mass spectrometry analysis was conducted by APTBIO (Shanghai, China). Briefly, the samples were first treated with reagents for reduction and alkylation, followed by enzymatic hydrolysis at 37°C for 20 h. The hydrolyzed samples were freeze-dried after desalting, and dissolved in 0.1% FA solution, which was ready for analysis or stored at –20°C. The samples were loaded onto a chromatographic trap column equilibrated with 95% liquid A (0.1% formic acid aqueous solution) for mass spectrometry analysis. The proteins in the samples were determined after the raw file of the mass spectrometry test (raw file) matched the data in the corresponding database via the search engine Proteome Discoverer 2.5. The search database parameters were as follows: enzyme (trypsin), database (UniProt_Homo_sapiens_203800_20220104), fixed modification [carbamidomethyl1 (C)], variable modification [carbamidomethyl1 (M)], maximum missing site of 2, and high confidence of the screened peptide. The mass spectrometry proteomics data have been deposited to the ProteomeXchange Consortium via the PRIDE partner repository with the dataset identifiers PXD047752 and PXD047753.

### Bioinformatics and statistical analyses

The KEGG terms were plotted by an online platform for data analysis and visualization ( https://www.kegg.jp). The false discovery rate was less than 0.05. GO terms were plotted by an online platform for data analysis and visualization ( https://www.bioinformatics.com.cn).

The statistical comparison of the results was performed using unpaired Student’s *t* tests with GraphPad Prism 10.0 software (GraphPad, La Jolla, USA) or Microsoft Excel software, and *P*<0.005 was considered to indicate statistical significance. For comparisons among more than two groups, one-way ANOVA was used. Unless otherwise stated, data are presented as the mean±standard deviation (SD) from at least three independent experiments.

## Results

### Expression of bait proteins and identification of proteins that interact with CHIKV nsP4

**Figure 1:**
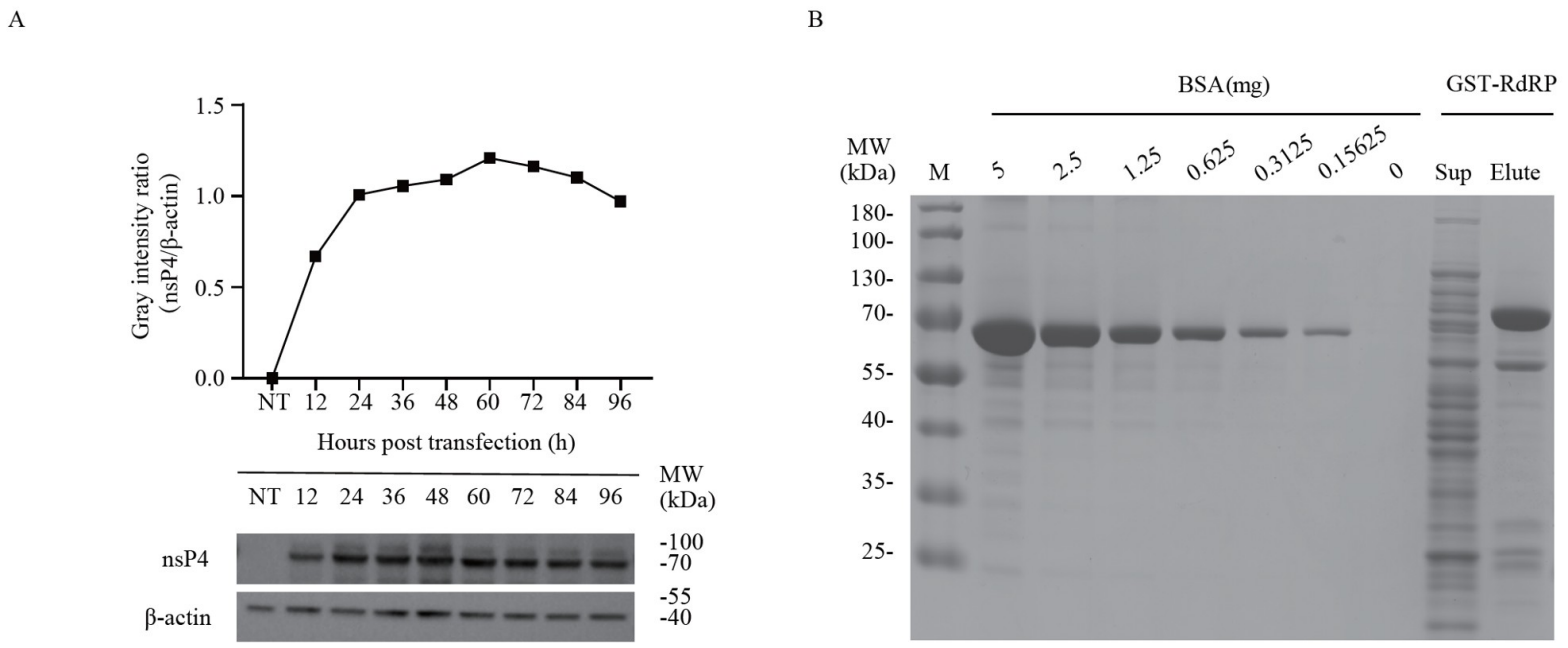
Figure 1 CHIKV nsP4 and RdRp were expressed in 293T cells and *E*. *coli* (A) Overexpression of CHIKV nsP4 in 293T cells. 293T cells in 6-well plates were transfected with recombinant pMD2.GΔVSV-nsP4. At the indicated time points post transfection, nsP4 in the cell lysate was analyzed with anti-nsP4 and anti-β-actin via western blot analysis. The intensity of each band in the bottom panel was analyzed by ImageJ software. The intensity of nsP4 was normalized to the intensity of β-actin and is plotted in the top panel by the software GraphPad Prism. (B) RdRp was expressed in E. coli and purified via a GST affinity column. SDS-PAGE was conducted to determine the purity and relative concentration of the RdRp. The supernatant (Sup) and eluted RdRp (Elute) were loaded on the right. Different amounts of BSA were loaded as a reference for relative quantitation of purified RdRp on the left.

To obtain bait proteins for capturing host interactors in Co-IP and GST pull-down experiments, full-length CHIKV *nsP4* with *flag* was cloned and inserted into the mammalian expression vector pMD2.GΔVSV and transfected into 293T cells for protein expression. Dynamic analysis revealed that the highest expression of nsP4 occurred at 60 h post transfection ( Figure 1A). Therefore, Co-IP experiments in this study were conducted on 293T cell lysates at 60 h post transfection. Given that the full-length CHIKV nsP4 is rarely soluble in *E*. *coli*, the core domain RdRp was cloned and inserted into the prokaryotic expression vector pGEX-6P-1 and expressed in *E*. *coli*. High-purity GST-fused RdRp was obtained after affinity purification ( Figure 1B). The yield of GST-RdRp was 0.5 mg per 1 liter of culture, which is sufficient for GST pull-down experiment.

### Host factors interacting with CHIKV nsP4 are involved in ubiquitin‒proteasome pathways

**Figure 2:**
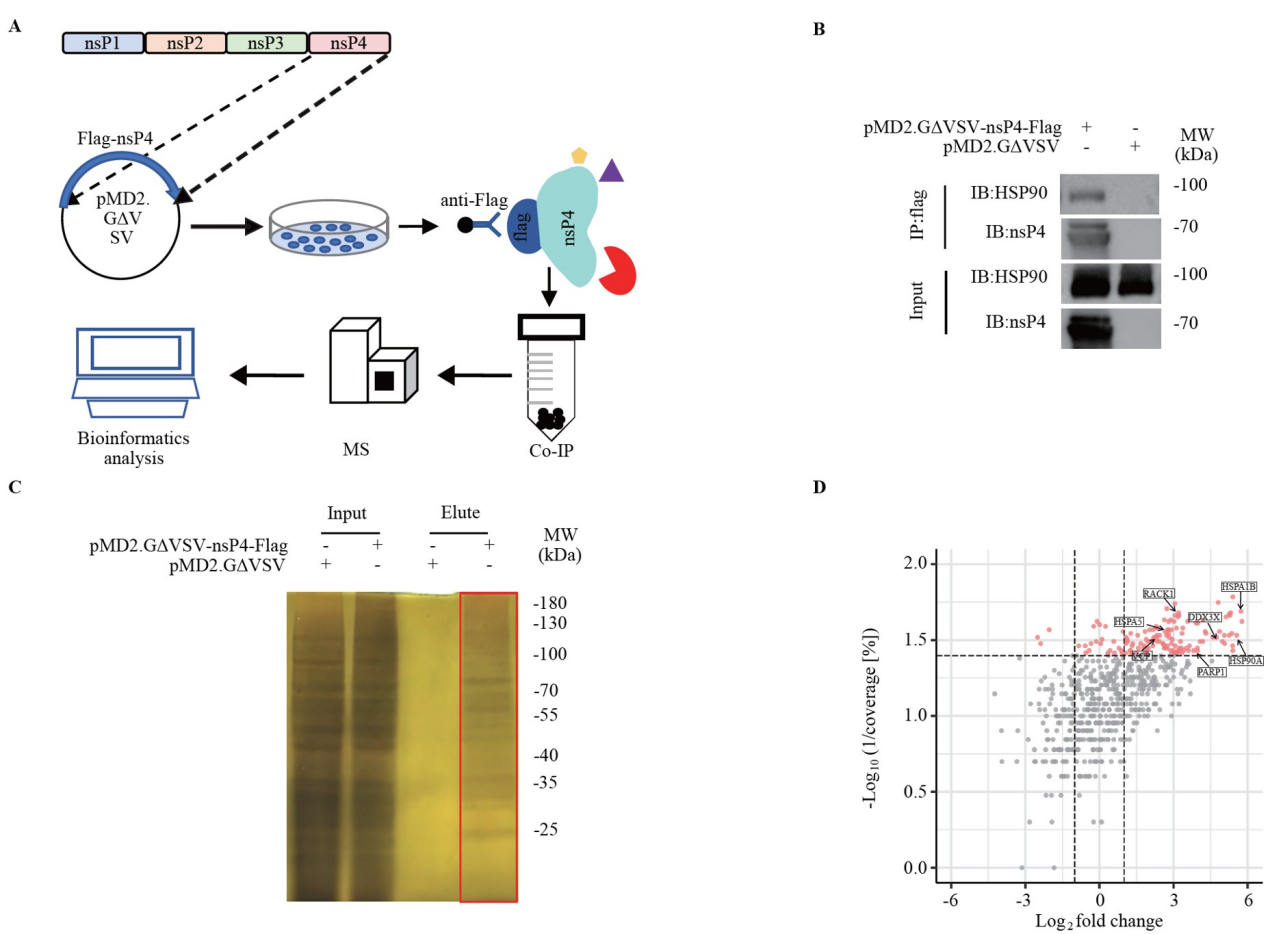
Figure 2 Host factors that interact with CHIKV nsP4 were identified by Co-IP (A) Overall pipeline for the integrated Co-IP-MS approach used to define host proteins that interact with CHIKV nsP4. (B) Detection of nsP4 and Hsp90 among the co-precipitated proteins. 293T cells in 6-well plates were transfected with 2.5 μg of Flag-nsP4 or the Flag empty vector for 60 h. Co-IP analysis was performed with anti-Flag magnetic beads, followed by western blot analysis with the indicated antibodies. (C) nsP4 co-precipitated proteins were separated by SDS-PAGE and visualized via silver staining. The gel in the box was cut for mass spectrometry analysis. (D) Volcano plot of candidate host factors obtained from Co-IP-MS. There were 611 proteins obtained from Co-IP-MS. Coverage [%]≥25, PSMs≥5, and unique peptides≥2 were set as the screening criteria. Proteins with a ratio (1/coverage [%]) <0.04 were defined as reliable proteins. Finally, 153 candidate host factors were screened via Co-IP-MS, and 7 representative proteins involved in the ubiquitin-proteasome pathway are highlighted with boxes. The X coordinate is the log 2fold change, and the Y coordinate is –log 10 (1/coverage [%]). The coverage of each protein was calculated as the ratio of the number of amino acids identified in a certain protein to the total number of amino acids identified in the protein with the highest score among all proteins. A higher coverage of the peptide represents a higher confidence of the identified protein. The fold change indicates the ratio of the relative abundance of a protein to the median number of proteins. The proteins with higher relative abundances are distributed on the right end of the X-axis, and the proteins with lower relative abundances are distributed on the left end of the X-axis. Each dot represents a protein. Red dots are reliable proteins with coverage [%]≥25. The gray dots are the unreliable proteins with coverage [%]<25.

To identify candidate host factors that interact with CHIKV nsP4, a co-immunoprecipitation mass spectrometry (Co-IP-MS) approach was used to identify cellular proteins that interact directly or indirectly with CHIKV nsP4 ( Figure 2A). Cell lysates without nsP4 overexpression were used as controls to exclude nonspecific proteins from all Co-IP-MS analysis. Consistent with previously reported results [28], Hsp90A in 293T cells co-precipitated with the CHIKV nsP4 protein, suggesting that the Co-IP system used in this study is reliable ( Figure 2B). As requested for mass spectrometry analysis, co-precipitated proteins were separated by SDS-PAGE and visualized by silver staining, which revealed the co-precipitation of nsP4 with cellular proteins ( Figure 2C). The gel containing the nsP4 co-precipitated proteins was cut after silver staining for MS analysis. A total of 993 proteins co-precipitated with nsP4 in 293T cells (accession number: PXD047752). To determine the confidence of the protein-protein interactions (PPIs), we first set PSMs≥5 and unique peptides≥2 as the screening criteria, which resulted in 661 proteins. A total of 153 proteins showed coverage [%]≥25, among which 17 proteins were associated with apoptosis or ubiquitination, including heat shock 90 kDa protein alpha (Hsp90Α), heat shock 70 kDa protein 1B (HSPA1B), poly [ADP-ribose] polymerase (PARP1), endoplasmic reticulum chaperone BiP (HSPA5), ATP-dependent RNA helicase (DDX3X), transitional endoplasmic reticulum ATPase valosin-containing protein (VCP), and receptor of activated protein C kinase 1 (RACK1) ( Figure 2D). KEGG and GO analyses also revealed that some of the 153 identified proteins are involved in ubiquitin unfolded proteolysis, proteasome activity and apoptosis ( Supplementary Figure S1A,B).

**Figure 3:**
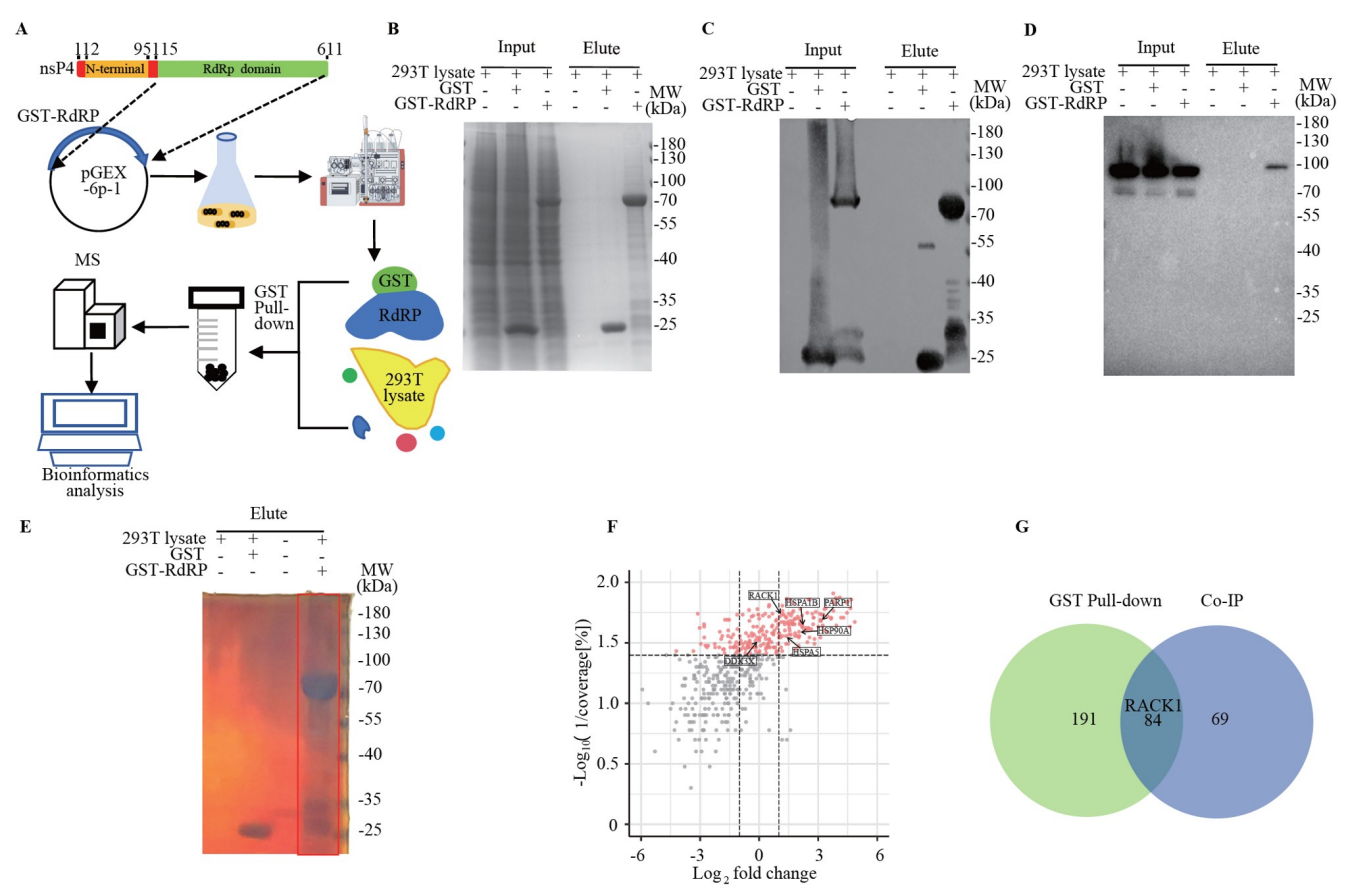
Figure 3 Identification of host factors interacting with CHIKV nsP4 by GST pull-down (A) Overall pipeline for the integrated GST pull-down-MS approach to define host proteins interacting with CHIKV nsP4 in 293T cells. (B) The RdRp domain of CHIKV nsP4 was expressed as a GST-fused protein in E. coli. Purified GST-RdRp or GST immobilized on GST-tag purification resin was incubated with the lysates of 293T cells. SDS-PAGE and Coomassie blue staining were used to examine the input and the eluate after the GST pull-down experiment. Western blot analysis was conducted to detect GST-associated proteins (C) and Hsp90 (D) in the input and eluted samples after the GST pull-down experiment. (E) SDS-PAGE and silver staining were performed to examine the eluate after the GST pull-down experiment. The gel in the box was cut for mass spectrometry analysis. (F) Candidate host factors obtained from GST pull-down-MS are shown as a volcano plot. Coverage [%]≥25, PSMs≥5, and unique peptides≥2 were set as the screening criteria. Proteins with a ratio (1/coverage [%]) < 0.04 were defined as reliable proteins. Finally, 275 candidate host factors are shown in this plot. The X coordinate was log 2-fold change, and the Y coordinate was –log 10 (1/coverage [%]). The coverage of each protein was calculated as the ratio of the number of amino acids identified in a certain protein to the total number of amino acids identified in the protein with the highest score among all proteins. A higher coverage of the peptide represents a higher confidence of the identified protein. The fold change indicates the ratio of the relative abundance of a protein to the median number of proteins. The proteins with higher relative abundances are distributed on the right end of the X-axis, and the proteins with lower relative abundances are distributed on the left end of the X-axis. Each dot represents a protein. Red dots are reliable proteins with coverage [%]≥25. The gray dots are the unreliable proteins with coverage [%]<25. (G) Venn diagram showing the overlapping proteins obtained by Co-IP-MS (blue) and GST pull-down-MS (green).

We further employed GST pull-down and mass spectrometry to confirm host factors that may interact with CHIKV nsP4 ( Figure 3A). Proteins pulled down by GST-RdRp were subjected to SDS-PAGE with Coomassie blue staining and western blot analysis with an anti-GST antibody ( Figure 3B,C). Notably, Hsp90 was also pulled down by the GST-RdRp fusion protein ( Figure 3D), which is consistent with the Co-IP results ( Figure 2B). As described for the Co-IP analysis, proteins pulled down by GST-RdRp were separated by SDS-PAGE and visualized via silver staining. A gel containing proteins pulled down by GST-RdRp was cut for mass spectrometry analysis ( Figure 3E). A total of 1574 proteins were identified among the proteins pulled down by GST-RdRp (accession number: PXD047753). To identify high-confidence protein-protein interactions (PPIs), we first set PSMs ≥5 and unique peptides≥2 as the screening criteria, which resulted in 509 proteins. A total of 275 proteins showed coverage [%]≥25. In addition to VCP, UBE2N and PSMB5, 14 proteins associated with apoptosis or ubiquitination according to Co-IP were also pulled down by the GST-RdRp fusion protein ( Figure 3F). There were 84 proteins identified by both GST pull-down and Co-IP, including RACK1 and other proteasome-associated proteins ( Figure 3G). Consistent with the results of Co-IP, KEGG and GO analysis showed that some of the 275 identified proteins are involved in ubiquitin unfolded proteolysis, proteasome and apoptosis ( Supplementary Figure S2A,B), including receptor for activated C kinase 1 (RACK1).

### Interruption of the interaction of RACK1 with nsP4 leads to a reduction in nsP4

**Figure 4:**
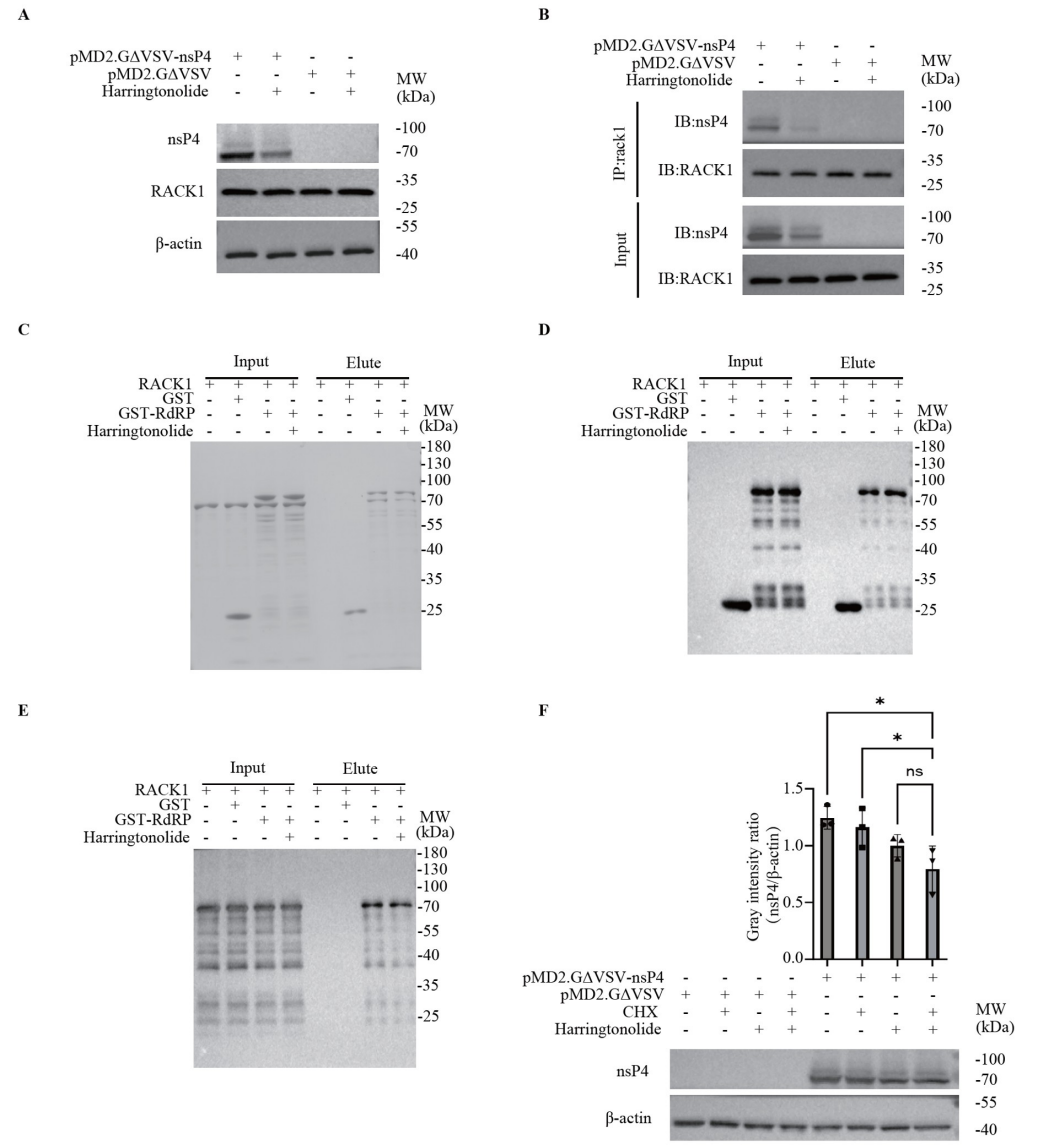
Figure 4 Inhibition of RACK1 results in a reduction in nsP4 (A) The RACK1 inhibitor harringtonolide reduced nsP4. 293T cells were transfected with the recombinant plasmid pMD2.GΔVSV-nsP4 or the vector pMD2.GΔVSV for 60 h, followed by treatment with the RACK1 inhibitor harringtonolide (25 μM) for 24 h. Western blot analysis was conducted to detect the indicated proteins with appropriate antibodies. (B) The RACK1 inhibitor harringtonolide reduced the amount of nsP4 bound to RACK1. 293T cells in 6-well plates were transfected with 2.5 μg of the recombinant plasmid pMD2.GΔVSV-nsP4 or the vector pMD2. GΔVSV for 60 h, followed by treatment with the RACK1 inhibitor harringtonolide (25 μM) for 24 h. Co-IP analysis was performed with anti-RACK1 magnetic beads. RACK1 and nsP4 were analyzed by western blot analysis. (C‒E) Assay of the direct interaction between RACK1 and RdRp. GST-RdRp or GST was incubated with purified RACK1 protein and resin with or without harringtonolide (25 μM) at 4°C overnight. GST elution buffer was used to resuspend the resin. The proteins pulled down by the GST-RdRp fusion protein in the supernatant were harvested for SDS-PAGE analysis (C), western blot analysis with an anti-GST antibody (D) or an anti-RACK1 antibody (E). (F) The reduction in nsP4 was caused by degradation but not inhibition of protein synthesis. 293T cells were transfected with the recombinant plasmid pMD2.GΔVSV-nsP4 or the vector pMD2.GΔVSV for 60 h and then treated with the RACK1 inhibitor harringtonolide (25 μM) or cycloheximide (CHX, 10 μg/mL) for 24 h, followed by western blot analysis (bottom panel). The gray intensity of each protein band was quantified via ImageJ software (top panel). The Y-axis represents the gray intensity ratio of nsP4 to β-actin. Data are presented as the mean±standard deviation (SD) from at least three independent experiments. ns, not significant; * P<0.05, ** P<0.01, *** P<0.001, **** P<0.0001.

Both Co-IP and GST pull-down experiments showed that CHIKV nsP4 co-precipitated with or pulled down RACK1, a scaffold protein involved in multiple cellular processes. This finding prompted us to ask what will happen if the interaction between nsP4 and RACK1 is interrupted. To answer this question, we treated nsP4-overexpressing 293T cells with the RACK1 inhibitor harringtonolide. No difference in cell viability was observed between the harringtonolide (25 μM)-treated group and the control group (data not shown). Notably, the level of overexpressed nsP4 was decreased in 293T cells after harringtonolide treatment ( Figure 4A), suggesting that RACK1 may be involved in the regulation of nsP4. It has been reported that harringtonolide inhibits the activity of RACK1 by competitively binding to RACK1 [29]. To further verify the interaction between nsP4 and RACK1, reverse Co-IP was conducted in 293T cells overexpressing nsP4 with anti-RACK1 magnetic beads. The results showed that RACK1 can precipitate with nsP4. Again, inhibition of this interaction led to a decrease in nsP4 ( Figure 4B), suggesting that RACK1 prevents the decrease in nsP4. We performed an *in vitro* GST pull-down assay with purified RACK1 and GST-RdRp, with or without the RACK1 inhibitor, revealing that GST-RdRp directly interacted with purified RACK1, which could be inhibited by the RACK1 inhibitor ( Figure 4C‒E). In CHX-treated 293T cells overexpressing nsP4, we observed a reduction in nsP4, which was worsened by the RACK1 inhibitor ( Figure 4F and Supplementary Figure S3). These results indicated that inhibiting the interaction between nsP4 and RACK1 reduces the level of nsP4 overexpression in 293T cells.

### RACK1 protects nsP4 from ubiquitin-mediated degradation

**Figure 5:**
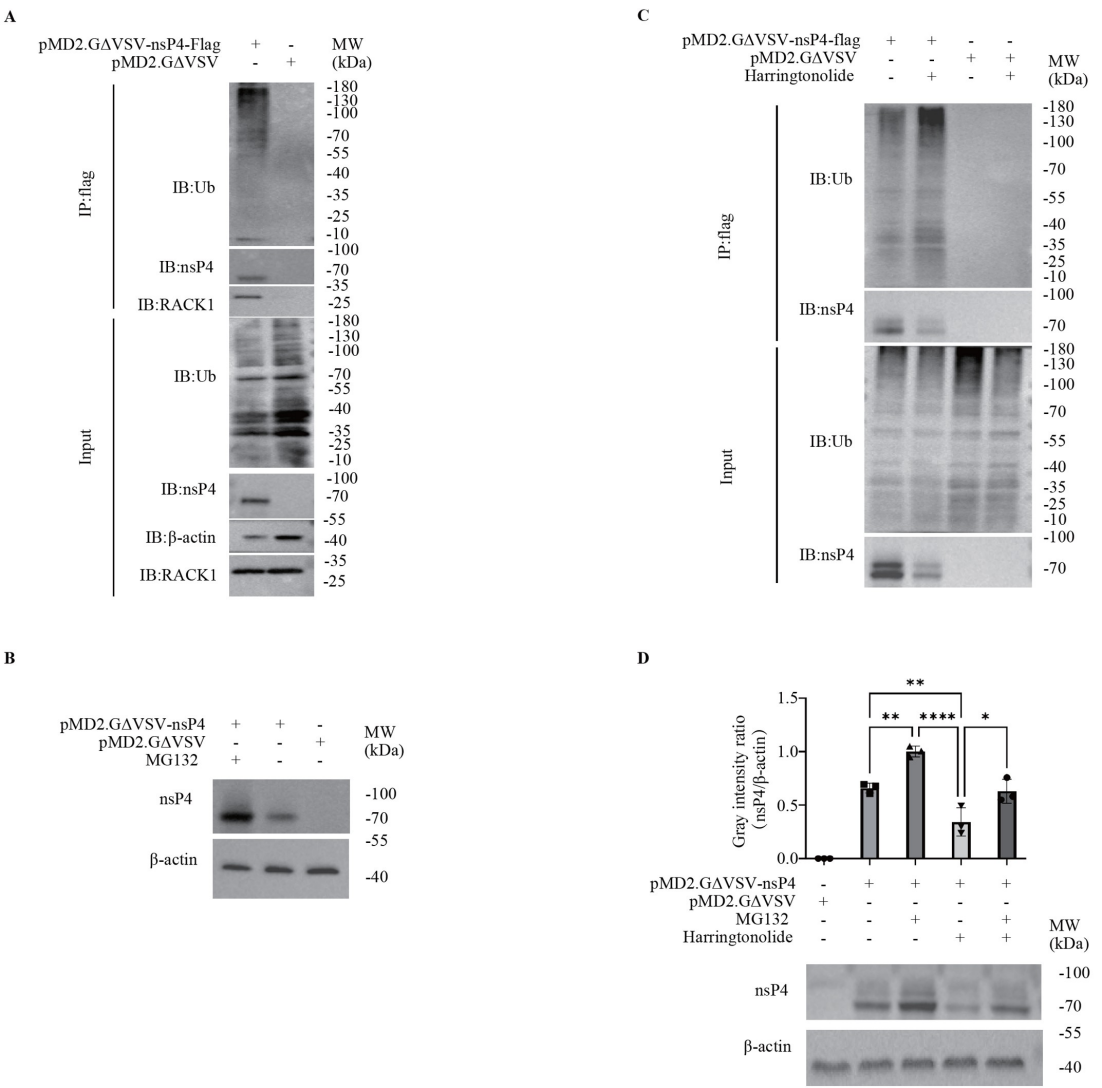
Figure 5 A proteasome inhibitor rescues the degradation of nsP4 induced by the RACK1 inhibitor (A) nsP4 is ubiquitinated in 293T cells. 293T cells in 6-well plates were transfected with 2.5 μg of the recombinant plasmid pMD2.GΔVSV-Flag-nsP4 or the vector pMD2.GΔVSV-Flag for 60 h, followed by Co-IP with anti-Flag magnetic beads. Ubiquitin, nsP4 and RACK1 were analyzed by western blot analysis. (B) Inhibition of the proteasome enhances the amount of nsP4. 293T cells were transfected with the recombinant plasmid pMD2. GΔVSV-nsP4 or the vector pMD2.GΔVSV for 60 h and then treated with the proteasome inhibitor MG132 (10 nM) for 24 h, followed by western blot analysis. (C) Inhibition of RACK1 increases the ubiquitination of nsP4. 293T cells in 6-well plates were transfected with 2.5 μg of the recombinant plasmid pMD2.GΔVSV-Flag-nsP4 or the vector pMD2.GΔVSV-Flag for 60 h and then treated with the RACK1 inhibitor harringtonolide (25 μM) for 24 h. Co-immunoprecipitation analysis and western blot analysis were performed with anti-Flag magnetic beads to detect the binding of nsP4 to ubiquitin. (D) The reduction in nsP4 induced by the RACK1 inhibitor can be reversed by the proteasome inhibitor. 293T cells were transfected with the recombinant plasmid pMD2.GΔVSV-nsP4 or the vector pMD2.GΔVSV for 60 h and then treated with the proteasome inhibitor MG132 (10 nM) or the RACK1 inhibitor harringtonolide (25 μM) for 24 h, followed by western blot analysis. The intensity of each band in the bottom panel was analyzed by ImageJ software. The intensity of nsP4 was normalized to the intensity of β-actin and is plotted in the top panel by the software GraphPad Prism. Data are presented as the mean±standard deviation (SD) from at least three independent experiments. ns, not significant; * P<0.05, ** P<0.01, *** P<0.001, **** P<0.0001.

Mass spectrometry analysis of proteins from Co-IP and GST pull-down revealed that nsP4 interactors are associated with ubiquitination and the proteasome pathway ( Supplementary Figure S1A,B). Therefore, we speculate that CHIKV nsP4 may be degraded via the ubiquitin‒proteasome pathway. Co-IP assays revealed that nsP4 co-precipitated with ubiquitin ( Figure 5A), suggesting that nsP4 is covalently modified by ubiquitin or that ubiquitin interacts with nsP4. Furthermore, inhibition of the proteasome by MG132 prevented the degradation of nsP4 in 293T cells ( Figure 5B), suggesting that nsP4 is degraded through the ubiquitin‒proteasome pathway. In contrast, inhibition of RACK1 by harringtonolide promoted nsP4 degradation and ubiquitination in 293T cells ( Figure 5C). Finally, the degradation of nsP4 induced by harringtonolide could be restored by the proteasome inhibitor MG132 ( Figure 5D and Supplementary Figure S3). Taken together, these results revealed that the host factor RACK1 protects nsP4 from degradation mediated by the ubiquitin-proteasome pathway.

## Discussion

It has been more than 70 years since CHIKF was first reported in the 1950s [30]. CHIKV has spread from the original tropical countries to subtropical and even template regions, including the Americas and Europe, and has attracted global attention [ 31‒ 33] . However, the pathogenesis of CHIKV remains to be defined due to insufficient knowledge about its molecular virology. At present, most of our knowledge about CHIKV molecular virology, particularly non-structural proteins, comes from the ECSA strains of CHIKV and other alphaviruses. Although the essential functions of nonstructural proteins are conserved among alphaviruses, a study of chimeric viruses showed that the interchange of nonstructural genes results in chimeric viruses with distinct biological properties from those of prototypic viruses, suggesting that they are not completely equivalent [34]. Therefore, other genotypic strains are necessary for the investigation of CHIKV to confirm some conclusions deduced from other viruses. In this study, the least studied protein, nsP4, from an Asian strain of CHIKV was overexpressed in 293T cells. Cellular interactions with CHIKV nsP4 were revealed via Co-IP and GST pull-down combined with high-throughput quantitative mass spectrometry. Notably, we identified a novel host interactor of RACK1 with CHIKV nsP4 and revealed that RACK1 protects CHIKV nsP4 from degradation by nsP4 through the ubiquitin-proteasome pathway. Therefore, RACK1 may be a potential target for the research and development of drugs against CHIKV.

CHIKV is categorized as a highly pathogenic alphavirus, and live CHIKV should be handled in the P3 laboratory, which hampers the study of CHIKV. Therefore, the CHIKV replicon, virus-like particles (VLPs) and pseudoviruses are alternatives widely used in exploring CHIKV molecular virology and pathogenesis [ 35‒ 37] . In addition, a specific viral protein used as a bait protein for yeast two-hybrid systems, Co-IP or GST pull-down, combined with MS, is commonly used to discover protein‒protein interactions and interacting domains [ 38‒ 40] . The advantage of this approach is that it avoids interference from other viral proteins with the target viral protein. Yeast two-hybrid screening with individual CHIKV viral proteins as bait proteins revealed 22 cellular proteins that interact with nsP2 or nsP4 [41]. Ten CHIKV proteins (nsP1-4, C, E3, E2, 6K, TF, and E1) individually overexpressed with a GFP tag in 293T cells were used to determine the cellular interactome of CHIKV [39]. The chaperone protein Hsp90 has been revealed to interact with overexpressed and GFP-tagged CHIKV nsP4 proteins [28]. TMEM45B, an IFN-stimulated gene product, has been shown to interact with CHIKV nsP1 and nsP4, possibly by blocking the formation of replication complexes and inhibiting viral replication [42]. Similarly, in this study, we identified the scaffold protein RACK1 as a novel interactor and potential regulator of nsP4. It will be interesting to determine the intracellular interaction between nsP4 and RACK1 in CHIKV-infected cells via colocalization experiments in the future.

The quantity of nsP4 is associated with alphavirus replication in cells [34]. However, the regulatory mechanism of nsP4 remains to be fully elucidated. There is an opal termination/stop codon located between the first ORF of the genes nsP3 and nsP4 at the 3′ terminus of the viral genome, which is one regulatory mechanism of nsP4 [ 43‒ 45] . Previous studies of SINV have shown that free nsP4 is unstable in cells and is degraded via the ubiquitin-dependent N-terminal pathway [46]. Consistently, in this study, we also showed that CHIKV nsP4 is ubiquitinated and degraded in cells, which is worsened by the inhibition of RACK1, suggesting that RACK1 is a novel potential regulator of CHIKV nsP4. In CHIKV-infected cells, treatment with proteasome inhibitors results in an increase in nsP4, while the viral titer decreases unexpectedly [47]. This may be because proteasome inhibitors increase the amount of antiviral proteins by blocking their degradation. It will be very interesting to study the effects of RACK1 inhibition on CHIKV replication.

RACK1, initially identified as a receptor for activated protein kinase C, is known to interact with constitutively bound and stimulus-dependent or transient proteins and is involved in several cellular processes related to physiological conditions and diseases [ 48, 49] . RACK1 functions as a scaffold protein by stabilizing the active or inactive confirmation of its partners and shuffling its partners to particular intracellular sites [50]. RACK1 has been frequently described to promote viral infection via various mechanisms, including immune escape [ 51, 52] , viral release [53], enhancement of viral replication [54] , cellular translation machinery [55], intracellular docking sites for viral proteins [56] , IRES-dependent viral translation [ 57, 58] , formation of viral replication organelles [50], induction of ribosome-based stress signaling [59], and inhibition of virus-induced apoptosis [60]. In contrast to its role in viral infection, RACK1 protects cells from infection by *Pasteurella multocida*, a gram-negative bacterium, via activation of the NLRP3 inflammasome [61]. In this study, we revealed that the inhibition of RACK1 interrupts the interaction with CHIKV nsP4 and promotes the degradation of viral nsP4 via the ubiquitin‒proteasome pathway. We hypothesize that RACK1 may promote CHIKV infection via its protection of nsP4, which remains to be tested in CHIKV-infected cells. After binding to its partners, RACK1 can modulate the enzymatic activities of its partners via the stabilization of their active or inactive conformation [ 62‒ 65] . Therefore, it will be important to determine whether RACK1 can affect the RdRp activity of CHIKV nsP4.

Hsp90, a highly abundant and ubiquitous chaperone protein, plays critical roles in the response to stress and maintenance of cellular homeostasis. In response to O _2_ stress, Hsp90 binds to the PAS-A domain of HIF-1α and prevents its degradation. In competition with Hsp90, RACK1 promotes the degradation of HIF-1α by recruiting ubiquitin ligases [66]. The host factor Hsp90 has been revealed to interact with CHIKV nsP4 and is involved in the assembly of the viral replication complex [28]. Consistently, in this study, we revealed the interactions of Hsp90 and RACK1 with CHIKV nsP4 by both Co-IP and GST pull-down ( Figures 2 and 3). It will be very interesting to study whether RACK1 could compete with Hsp90 for binding to CHIKV nsP4.

CHIKV nsP4, with a length of 611 amino acids, contains two domains. The N-terminal domain consists of 100 amino acids, which is essential for nsP4 function and is associated with viral adaptation to host cells [67]. The C-terminal domain contains the core RdRp domain, covering aa 151‒599 of CHIKV nsP4. In this study, we used the RdRp domain of CHIKV nsP4 to pull down RACK1, which indicated that RdRp contains a binding site for RACK1. Defining the key residues of nsP4 associated with the interaction with RACK1 is highly important. On the other hand, the key domains involved in the interaction of RACK1 with nsP4 should also be further determined by overexpressing RACK1 mutants in RACK1-deficient cells.

**Figure 6:**
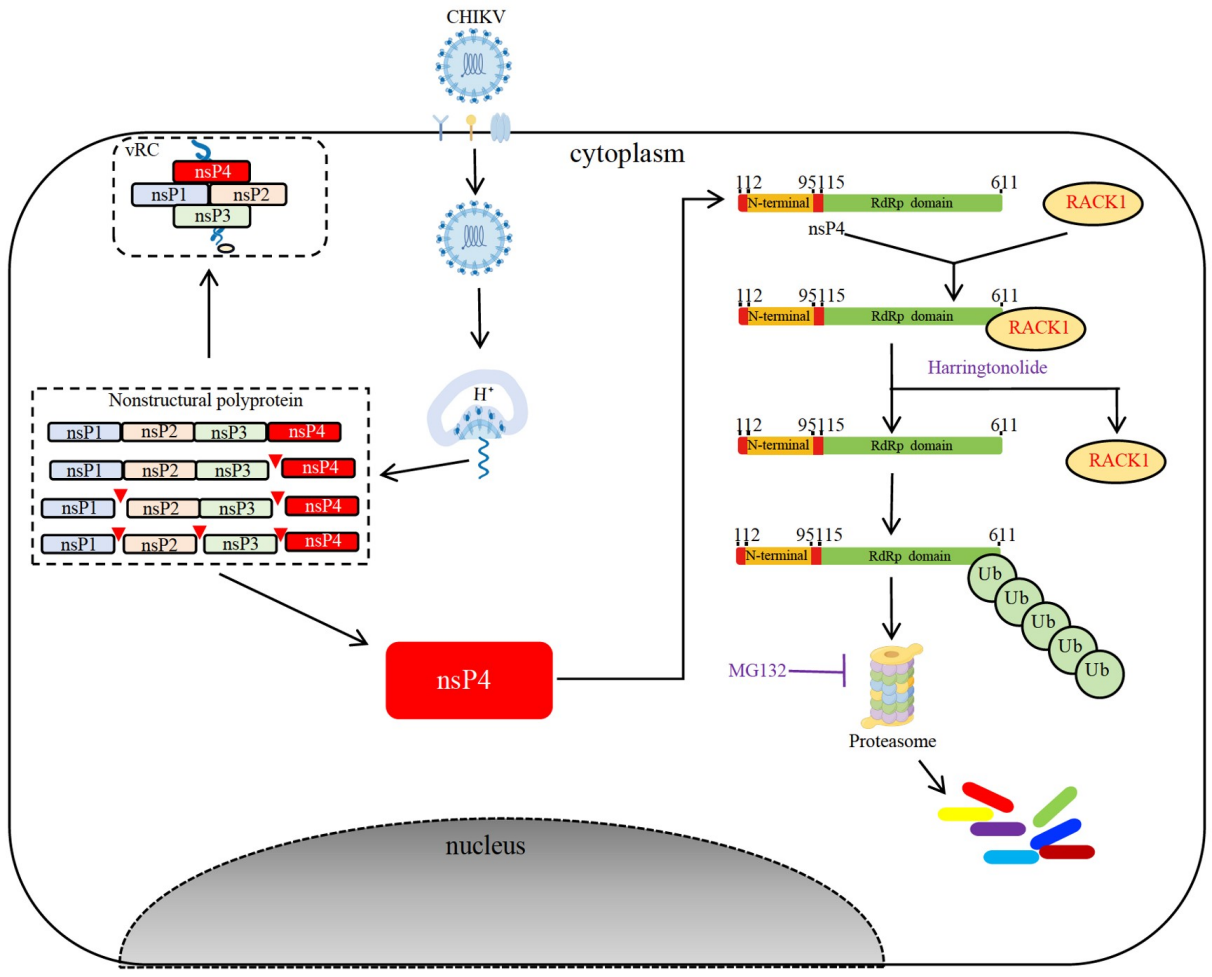
Figure 6 A proposed model for RACK1 regulation of CHIKV nsP4 in host cells After being cleaved from nonstructural polyproteins, CHIKV nsP4 exists in two forms: free nsP4 and a component of the viral replication complex (vRC) with other nonstructural proteins. Free nsP4 recruits RACK1 to protect it from degradation through the ubiquitin‒proteasome pathway. The competitive inhibition of RACK1 by harringtonolide promotes the degradation of nsP4, which can be restored by the proteasome inhibitor MG132.

In summary, this study identified RACK1 as a novel interactor of CHIKV nsP4 and revealed a possible mechanism by which the host protein RACK1 regulates CHIKV nsP4. After entry into the host cell, CHIKV genomic RNA is released into the cytoplasm as a template for synthesis of the nonstructural polyprotein. The polyprotein is processed by self-enzymatic activity to produce intermediate or individual nonstructural proteins that are essential components of the viral replication complex. NsP4, the first protein cleaved from the polyprotein, functions as the enzyme RdRp and plays critical roles in viral RNA replication. In addition, free nsP4 interacts with RACK1 to escape degradation through the ubiquitin‒proteasome pathway ( Figure 6). The interaction between RACK1 and nsP4 is a potential target for the development of antiviral drugs. It is necessary to determine whether nsP4 is commonly affected by all alphaviruses.

## Supplementary Data

Supplementary data is available at *Acta Biochimica et Biophysica Sinica* online.
